# BJ-B11, an Hsp90 Inhibitor, Constrains the Proliferation and Invasion of Breast Cancer Cells

**DOI:** 10.3389/fonc.2019.01447

**Published:** 2019-12-18

**Authors:** Kaisheng Liu, Juan Chen, Fang Yang, Zhifan Zhou, Ying Liu, Yaomin Guo, Hong Hu, Hengyuan Gao, Haili Li, Wenbin Zhou, Bo Qin, Yifei Wang

**Affiliations:** ^1^Shenzhen People's Hospital, The First Affiliated Hospital of Southern University of Science and Technology, The Second Clinical Medical College of Jinan University, Shenzhen, China; ^2^Shenzhen Nanshan District Shekou People's Hospital, Shenzhen, China; ^3^Institute of Biomedicine, College of Life Science and Technology, Jinan University, Guangzhou, China

**Keywords:** breast cancer, Hsp90, BJ-B11, proliferation, invasion, migration, EMT

## Abstract

Breast cancer is the leading cause of cancer-related deaths in women; however, its underlying etiology remains largely unknown. In this study, we systematically analyzed breast cancer tissues using comprehensive iTRAQ labeled quantitative proteomics, identifying 841 differentially expressed proteins (474 and 367 significantly over- and under-expressed, respectively), which were annotated by protein domain analysis. All the heat shock proteins identified were upregulated in breast cancer tissues; Hsp90 upregulation was also validated by RT-qPCR and immunohistochemistry, and high Hsp90 protein levels correlated with poorer survival. Hsp90AA1 overexpression promoted MDA-MB-231 cell proliferation, whilst BJ-B11, an Hsp90 inhibitor, hampered their invasion, migration, and proliferation in a time and dose-dependent manner and induced cell cycle arrest and apoptosis. BJ-B11 inhibited the expression of epithelial-mesenchymal transition (EMT) marker in MDA-MB-231 cells, whereas Hsp90AA1 promoted its expression. Moreover, BJ-B11 inhibited tumor growth in xenograft model. Altogether, Hsp90 activation is a risk factor in breast cancer patients, and BJ-B11 could be used to treat breast cancer.

## Introduction

Breast cancer is the most frequently diagnosed cancer in women (1), and its incidence has increased in most developing countries over the past few decades (1, 2). Although patients with breast cancer have a high 5-year survival rate following treatment, the survival rate decreases rapidly for patients with more advanced disease (3–5). Triple-negative breast cancers (TNBCs) account for 15% of all breast cancers and lack estrogen, progesterone, and ERBB2 receptor expression (6). TNBCs are poorly differentiated, and there are no specific treatment guidelines for this breast cancer subgroup (7, 8); therefore, biomarkers and more effective medical therapies are urgently required.

Breast cancer is coordinately controlled by regulatory networks; thus, understanding these networks could help identify candidates for the diagnosis, prediction, and therapy of breast cancer. Proteomics approaches are often used to acquire a comprehensive and quantitative profile of protein expression. Isobaric tags for relative and absolute quantitation (iTRAQ) is a novel and unbiased approach to simultaneously quantify relative protein abundance (9); in particular, the method enables protein quantification during various developmental stages (10).

Hsp90 is highly expressed in various cancers (11). It is responsible for the stability and function of client proteins, including Akt, IKKα, B-Raf, and GSK3β, which are critical for cell survival and proliferation (12). Therefore, Hsp90 is a potential therapeutic target and diagnostic marker for cancer (13, 14). BJ-B11 is a novel Hsp90 inhibitor that reportedly exhibits antitumor activity in myeloid leukemia and esophageal carcinoma (15, 16); however, its antitumor activity in breast cancer has not yet been investigated.

In this study, we investigated whether Hsp90 was associated with breast cancer and whether BJ-B11 affected the functions of breast cancer cells. Our findings suggested that Hsp90 could be a candidate for the early diagnosis, prognosis, and therapy of breast cancer and that BJ-B11 could be used to treat breast cancer.

## Materials and Methods

### Primary Breast Cancer Samples

Tumor tissue and adjacent normal tissue samples were collected at the Department of Thyroid and Breast Surgery, Shenzhen People's Hospital, with the informed consent of the patients. The study (LL-KT-2015002) was approved by the Ethics Committee of the hospital. The clinicopathological information regarding the samples is detailed in the Table S1. Tumor tissues and normal tissues (10 mg) were homogenized for proteomics and iTRAQ labeling followed by LC-MS/MS analysis. The protein with iTRAQ ratio (tumor tissue/normal tissue) <0.83 or > 1.2 (*P* < 0.05) was considered to be significantly differentially expressed.

### Cell Culture and Reagents

The human breast cancer cell line MDA-MB-231 was obtained from the American Type Culture Collection (Manassas, VA, USA) and cultured in DMEM/F12 supplemented with 10% FBS, 100 μg/mL streptomycin, and 100 unit/mL penicillin in a humidified incubator in a 5% CO_2_ atmosphere at 37°C.

BJ-B11 was prepared in our lab, as previously described (17), and the 10 mmol/L BJ-B11 stock solution in DMSO was stored at 4°C. Plasmids expressing wild-type Hsp90AA1 were provided by SAGENE (Guangzhou, Guangdong, China). Mouse anti-E-cadherin (cat: 14472), rabbit anti-vimentin (cat: 3932), and mouse anti-β-actin (cat: 3700) antibodies were purchased from CST (MA, USA).

### Cell Viability and Apoptosis Assay

CCK-8 (Dojindo, Japan) was used to detect cell viability. Cell apoptosis induced by BJ-B11 was determined using AnnexinV/PI (KeyGEN, Nanjing, China) staining, followed by flow cytometry (Beckman Coulter, CA, USA) according to the manufacturer's instructions.

### Cell Cycle Analysis

Cells were treated with BJ-B11 for 48 h, harvested in cold PBS, fixed in 70% ethanol, and stored overnight at 4°C. The cells were then washed twice with cold PBS, resuspended in 50 μg/mL PI staining reagent containing 100 μg/mL RNase and 0.1% Triton X-100 for 30 min in the dark, and analyzed by flow cytometry (Becton-Dickinson, CA, USA).

### Real-Time Quantitative Polymerase Chain Reaction (RT-qPCR)

Total RNA was extracted using TRIZOL (Thermo Fisher Scientific) and subjected to qRT-PCR using the primers shown in Table S2). Gene expression was normalized against GAPDH using the relative ^ΔΔ^CT method and is reported as relative expression compared to the control.

### Cell Invasion Assay

A total of 2 × 10^4^ MDA-MB-231 cells treated with or without BJ-B11 were added to Transwell inserts and cultured in an incubator for 16 h. Cells inside the insert were cleaned thoroughly with a cotton swab, while those on the underside were fixed in 4% paraformaldehyde for 5 min and stained with 0.5% crystal violet solution. At least five random fields were counted per insert, and each group consisted of three replicates.

### Tissue Microarray

Human breast tissue (HBreD077Su01, Shanhai Xinchao, China) and breast cancer tissue (HBreD140Su05, Shanhai Xinchao, China) microarrays consisting of 77 adjacent non-malignant tissue samples and 140 breast cancer tissue samples, were stained with rabbit anti Hsp90 (4874, CST, USA). Immunohistochemical staining was carried out according to the manufacturer's instructions. Slides were evaluated for their positive staining rate (0, negative; 1, 1–25%; 2, 26–50%; 3, 51–75%; and 4, 76–100%) and the staining intensity of the positively stained cells (0, none; 1, weak; 2, moderate; and 3, strong). Samples were grouped according to the H score, which was the product of the “staining intensity” and “staining positive rate” scores: low expression group, <8; and high expression group, ≥ 8. Two investigators evaluated each tissue section independently.

### The Cancer Genome Atlas (TCGA) Data Analysis

The expression level and survival of Hsp90AA1 and Hsp90AB1 in breast cancer were analyzed using the UALCAN platform.

### Xenograft Model

Female BALB/c Nude Mice (6-week-old) were obtained from the Guangdong Medical Laboratory Animal Center. They were maintained in an air-conditioned room with controlled temperature of 21 ± 2°C, and humidity of 30–70% in a 12 h light/darkness cycle regulation and were fed laboratory chow and water *ad libitum*. All animal experiments were approved by the Animal Ethics Committee of Shenzhen People's Hospital (No. LL-KY-2019512). The athymic female nude mice were injected with about 5 × 10^6^ MDA-MB-231 cells subcutaneously. When the tumors were measurable, the mice were randomly assigned into treatment group receiving oral BJ-B11 (20 mg/kg, daily for a total of 27 days) or the control group receiving oral vehicle alone. All mice were euthanized at day 28, and the tumors were excised and weighed to evaluate tumor growth inhibition.

### Statistical Analysis

Data are presented as the mean ± SD, and Student's unpaired *t*-tests were used for the statistical analysis of differences between two groups. Differences between groups were analyzed using Prism 6 (GraphPad, Inc., San Diego, CA). *P* < 0.05 were considered statistically significant.

## Results

### Identification of Hsp90 as a Diagnostic Marker

**Figure 1:**
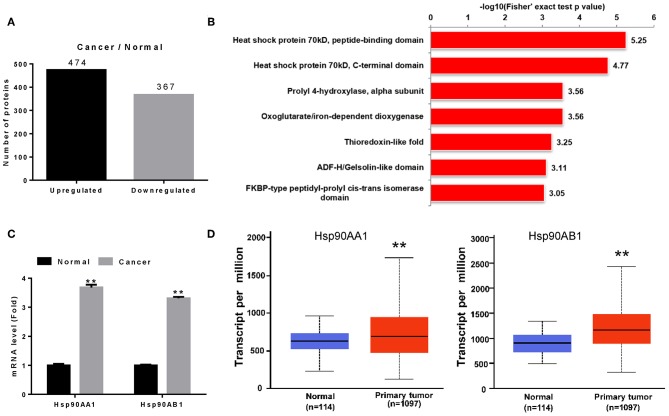
Identification of Hsp90 as a diagnostic marker. **(A)** Differentially expressed proteins in breast cancer. **(B)** Differentially expressed proteins were classified according to their protein domains (*P* < 0.001). **(C)** Validation of Hsp90AA1 and Hsp90AB1 mRNA levels in healthy and breast cancer tissues by RT-qPCR. **(D)** Hsp90AA1 and Hsp90AB1 mRNA levels in healthy and breast cancer tissues from TCGA database (^**^P < 0.01).

We screened proteins that were differentially expressed between tumor tissues and adjacent normal tissues using iTRAQ with ratio (tumor:adjacent normal tissue) thresholds of >1.2 or <0.83, which indicated higher or lower protein expression in tumor tissue than in adjacent normal tissue, respectively. A total of 841 differentially expressed proteins were identified, among which 474 and 367 were up- and down-regulated in breast cancer tissues (Figure 1A).

To understand the functions of these differentially expressed proteins, they were classified according to their protein domains (*P* < 0.001; Figure 1B). Heat shock proteins showed the most significant change of all the upregulated domains, with Hsp90AA1, Hsp90AB1, TRAP1, HspA5, HspB1, HspE1, HspD1, HspA1B, HspA8, HspA9, and HspA4 identified in breast cancer tissue (Table S3). RT-qPCR revealed that Hsp90AA1 and Hsp90AB1 mRNA levels increased in breast cancer (Figure 1C), consistent with TCGA data (Figure 1D). These results suggest that the highly expressed Hsp90 could act as a diagnostic marker for breast cancer.

### Identification of Hsp90 as a Prognostic Marker

**Figure 2:**
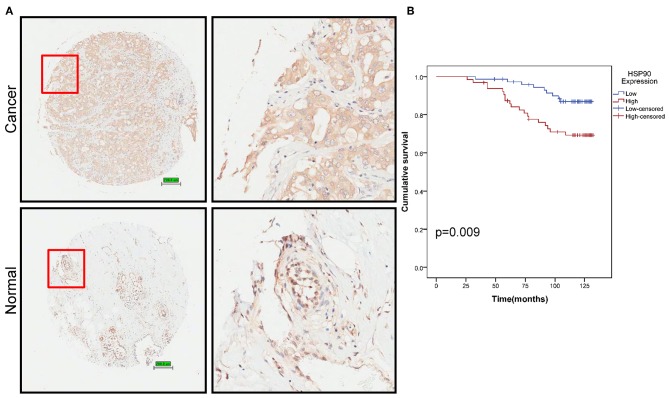
Identification of Hsp90 as a prognostic marker. **(A)** Immunohistochemical staining of Hsp90 in normal (*n* = 77) and breast cancer (*n* = 140) tissues. Representative images are shown. Scale bar, 200 μm. **(B)** Cumulative survival of breast cancer patients with low or high Hsp90 expression.

We then performed tissue microarray analysis, finding that Hsp90 protein levels increased in breast cancer tissue (*P* = 0.014; Figure 2A and Table S4). Moreover, Hsp90 expression was significantly correlated with clinical tumor grade (Table S5); therefore, we analyzed the survival rate of patients with breast cancer. High Hsp90 levels predicted poor survival (Figure 2B), consistent with TCGA data (Figure S1). Thus, the univariate and multivariate analyses suggested that Hsp90 could serve as a prognostic indicator for breast cancer (Table S6).

### BJ-B11 Induces Apoptosis and Cell Cycle Arrest in Breast Cancer Cells

**Figure 3:**
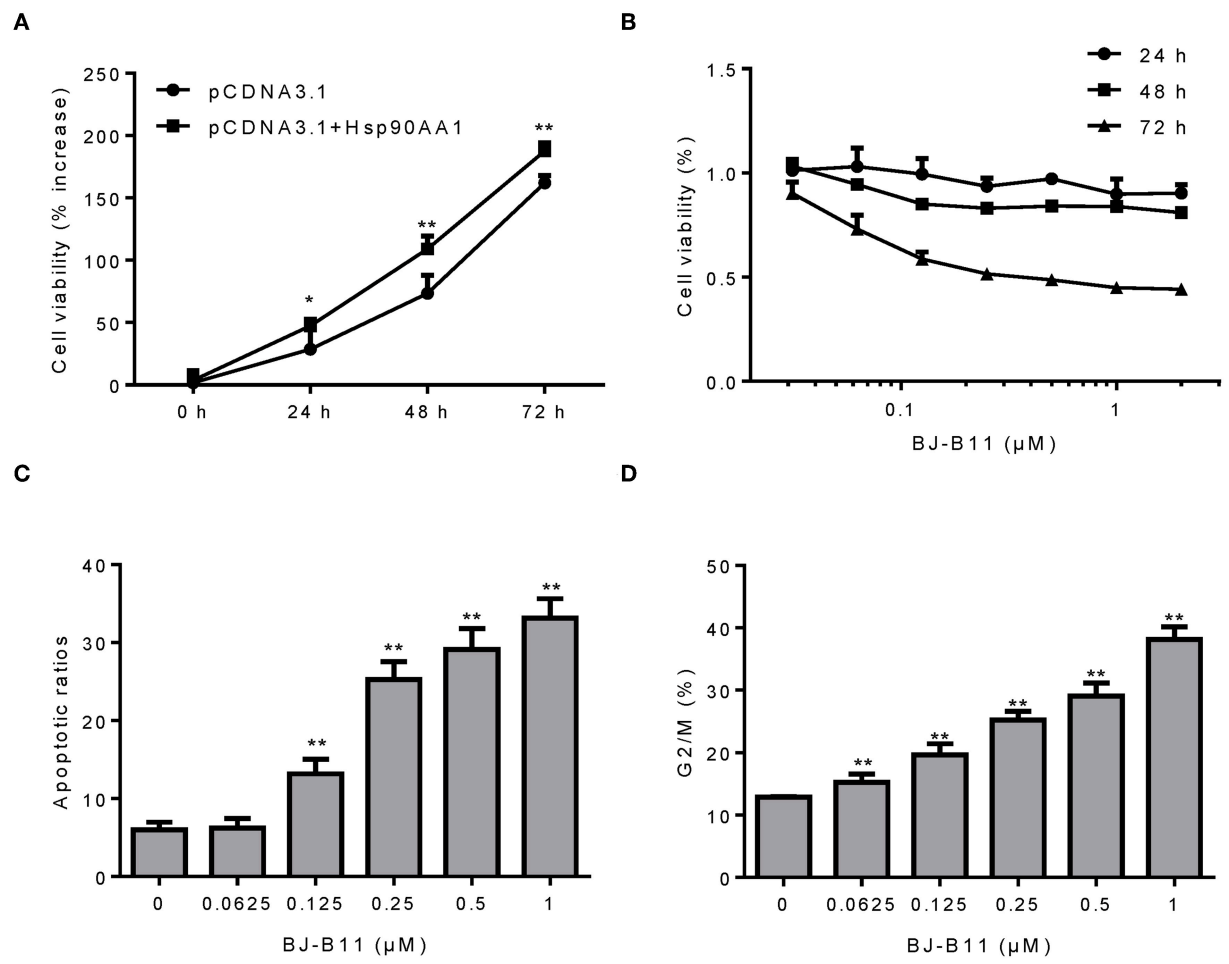
BJ-B11 induces apoptosis and cell cycle arrest in breast cancer cells. **(A)** The viability of MDA-MB-231 cells overexpressing Hsp90AA1 was tested by CCK8 after 24, 48, and 72 h (^*^P < 0.05, ^**^P < 0.01). **(B)** The viability of cells treated with the indicated concentrations of BJ-B11 for 24, 48, or 72 h was tested by CCK8. **(C)** Cells were treated with BJ-B11 for 48 h, collected, and stained with AnnexinV/PI. Apoptotic cells were analyzed by flow cytometry (^**^P < 0.01). **(D)** Cells were treated with BJ-B11 for 48 h, collected, fixed, and stained with PI. The cell cycle stage was analyzed by flow cytometry (^**^P < 0.01).

Since our findings suggested that Hsp90 was involved in breast cancer, we examined whether manipulating the Hsp90 gene affected the proliferation of breast cancer cells. Hsp90AA1 overexpression increased MDA-MB-231 cell proliferation (Figure 3A), whereas treating the cells with BJ-B11, an Hsp90 inhibitor, for 24, 48, or 72 h inhibited their growth in a time- and dose-dependent manner (Figure 3B) and induced dose-dependent apoptosis (Figure 3C) and G2/M cell cycle arrest (Figure 3D). Taken together, Hsp90AA1 promoted the proliferation of breast cancer cells, whilst BJ-B11 could be used to treat breast cancer.

### BJ-B11 Inhibited Invasion and Migration of Breast Cancer Cells

**Figure 4:**
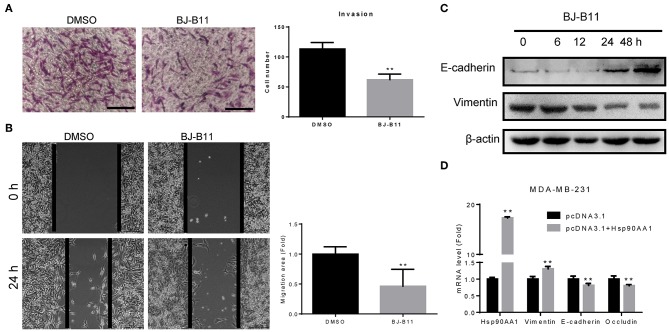
BJ-B11 inhibited the invasion and migration of breast cancer cells. **(A)** Cells were cultured in Transwell inserts for 16 h with or without 0.0625 μM BJ-B11 and then stained with crystal violet. Cell number was calculated and analyzed (^**^P < 0.01). Scale bar, 100 μm. **(B)** Wound healing assay of MDA-MB-231 cell treated with or without 0.0625 μM BJ-B11 for 24 h (^**^P < 0.01). Scale bar, 200 μm. **(C)** Western blot assay of MDA-MB-231 cells treated with 0.0625 μM BJ-B11 for the indicated length of time. **(D)** MDA-MB-231 cells overexpressing Hsp90AA1 were cultured for 48 h. Hsp90AA1, E-cadherin, vimentin, and occludin mRNA levels were tested by RT-qPCR (^**^P < 0.01).

MDA-MB-231 cells were cultured with or without BJ-B11 in Transwell inserts for 16 h. BJ-B11 inhibited the invasion of the MDA-MB-231 cells (Figure 4A) and inhibited Hsp90, thus significantly suppressing MDA-MB-231 cell migration (Figure 4B). We also analyzed epithelial-mesenchymal transition (EMT) markers related to invasion and migration, finding that BJ-B11 upregulated E-cadherin and downregulated vimentin (Figure 4C). Conversely, Hsp90AA1 overexpression upregulated vimentin and downregulated E-cadherin and occludin (Figure 4D), suggesting that Hsp90 plays a vital role in EMT in breast cancer. Taken together, these data strongly suggest that BJ-B11 inhibits cell invasion and migration by affecting EMT in breast cancer.

### BJ-B11 Inhibited Tumor Growth *in vivo*

**Figure 5:**
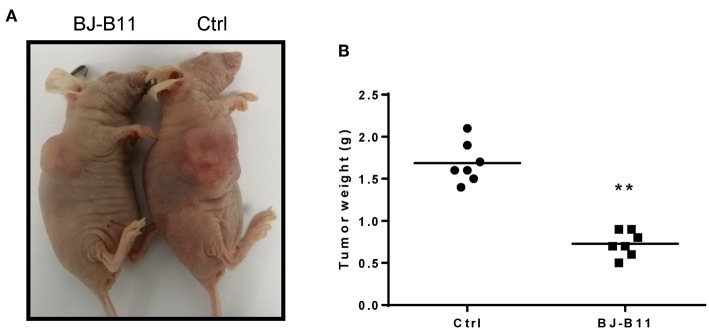
BJ-B11 inhibited tumor growth *in vivo*. **(A)** Representative picture of the xenograft model. **(B)** Analysis of tumor weight of BJ-B11 treated group and control group (*n* = 7, ^**^P < 0.01).

We further tested whether BJ-B11 could suppress cell growth of breast cancer *in vivo*. First, we established xenograft models by subcutaneous injection of MDA-MB-231 cells into the right flanks of mice. We then tested the anti-tumor effects of BJ-B11 on MDA-MB-231 cancer cells. Nude mice bearing MDA-MB-231 tumor xenografts were treated with 20 mg/kg BJ-B11 (*n* = 7) or physiological saline (*n* = 7) for 27 days. Bodyweight was measured each day before the administration of BJ-B11. Results showed that BJ-B11 inhibited tumor growth significantly *in vivo* (Figures 5A,B).

## Discussion

This study investigated breast cancer proteome using iTRAQ to obtain a global view and identify therapeutic targets for breast cancer. We identified 841 differentially expressed proteins and showed that heat shock proteins could be candidate biomarkers for the early diagnosis and therapy of breast cancer. Moreover, this is the first study to investigate the antitumor activity of BJ-B11 in TNBCs, showing that BJ-B11 could be used to treat breast cancer.

Multiple heat shock proteins, including Hsp90AA1, Hsp90AB1, TRAP1, HspA5, HspB1, HspE1, HspD1, HspA1B, HspA8, HspA9, and HspA4, were significantly upregulated in breast cancer tissue. Hsp70, HspA8, HspA9, HspA5, and Hsp110s may constitute up to 3% of the total protein in unstressed human cells (18). Moreover, Hsp70 is upregulated in various human cancers and associated with tumorigenesis (19, 20). HspA8 is overexpressed in cancer cells and it belongs to the Hsp70 family (21). Its depletion in RL-95-2 and HEC-1B cells was shown to suppress cell growth and promote apoptosis, suggesting that HspA8 could be a candidate biomarker for endometrial carcinoma (22).

Hsp90 is an ATP-dependent molecular chaperone (23). Reportedly, Hsp90 expression levels are associated with disease progression and survival in melanoma, gastrointestinal stromal tumors and non-small cell lung cancer (24, 25). Moreover, high Hsp90 expression has been associated with decreased survival in breast cancer (26) whilst its inhibition can suppress growth and promote apoptosis in breast cancer cells, suggesting that Hsp90 could act as both a biomarker and a therapeutic target for breast cancer (27). A phase II study of 17-AAG in breast cancer showed that Hsp90 inhibitors exhibit significant anticancer activity (28). Previously, we demonstrated that the Hsp90 inhibitor SNX-2112 suppressed MCF-7 cell proliferation and induced apoptosis (27), whilst the Hsp90 inhibitor PU-H71 has been shown to induce a complete response in TNBC models (29). BJ-B11 is a novel Hsp90 inhibitor that can inhibit cancer cell proliferation and exhibits anti-HSV activity (15, 16). To the best of our knowledge, this is the first study to investigate the antitumor activity of this molecule in TNBCs; BJ-B11 inhibited the proliferation, invasion, and migration of breast cancer cells, which may be associated with EMT. In addition, BJ-B11 showed significant antitumor activity *in vivo*. However, the underlying mechanism requires further clarification.

In summary, our study provides new insights into the molecular changes that occur in breast cancer. Hsp90AA1, Hsp90AB1, TRAP1, HspA5, HspB1, HspE1, HspD1, HspA1B, HspA8, HspA9, and HspA4 were validated as potential biomarkers for breast cancer tissue. In particular, Hsp90 plays important roles in breast cancer development and could be a candidate biomarker for the early diagnosis and therapy of breast cancer. Moreover, BJ-B11 could open new, potential therapeutic alternatives for breast cancer.

## Data Availability Statement

The datasets generated for this study are available on request to the corresponding author.

## Ethics Statement

The studies involving human participants were reviewed and approved by Ethics Committee of Shenzhen People's Hospital. The patients/participants provided their written informed consent to participate in this study. The animal study was reviewed and approved by Ethics Committee of Shenzhen People's Hospital.

## Author Contributions

KL conceived, designed the experiments, and wrote the manuscript. KL, YL, and YG performed the experiments and analyzed the data. KL, JC, FY, ZZ, HH, HG, HL, WZ, BQ, and YW contributed reagents, materials, and analysis tools.

### Conflict of Interest

The authors declare that the research was conducted in the absence of any commercial or financial relationships that could be construed as a potential conflict of interest.

## Supplementary Material

Figure S1 Cumulative survival analysis of breast cancer patients with low or high Hsp90AA1 **(A)** or Hsp90AB1 **(B)** expression.

Table S1 Sample information.

Table S2 Primers used in this study.

Table S3 Hsp family members upregulated in breast cancer tissue.

Table S4 Differential Hsp90 expression in healthy and breast cancer tissues.

Table S5 Correlation between Hsp90 expression and clinicopathological characteristics.

Table S6 Univariate and multivariate analyses of factors correlated with the overall survival of breast cancer patients.

## Abbreviations

Hsp90: heat shock protein 90
TNBC: triple-negative breast cancer
iTRAQ: isobaric tags for relative and absolute quantitation
GO: Gene Ontology
RT-qPCR: real-time quantitative polymerase chain reaction
GAPDH: glyceraldehyde-3-phosphate dehydrogenase
EMT: epithelial-mesenchymal transition.
