# Supplementary figures and images for: BJ-B11, an Hsp90 Inhibitor, Constrains the Proliferation and Invasion of Breast Cancer Cells
Source: Front Oncol. 2019 Dec 18;9:1447. doi: 10.3389/fonc.2019.01447 (PMC6930179; doi:10.3389/fonc.2019.01447)

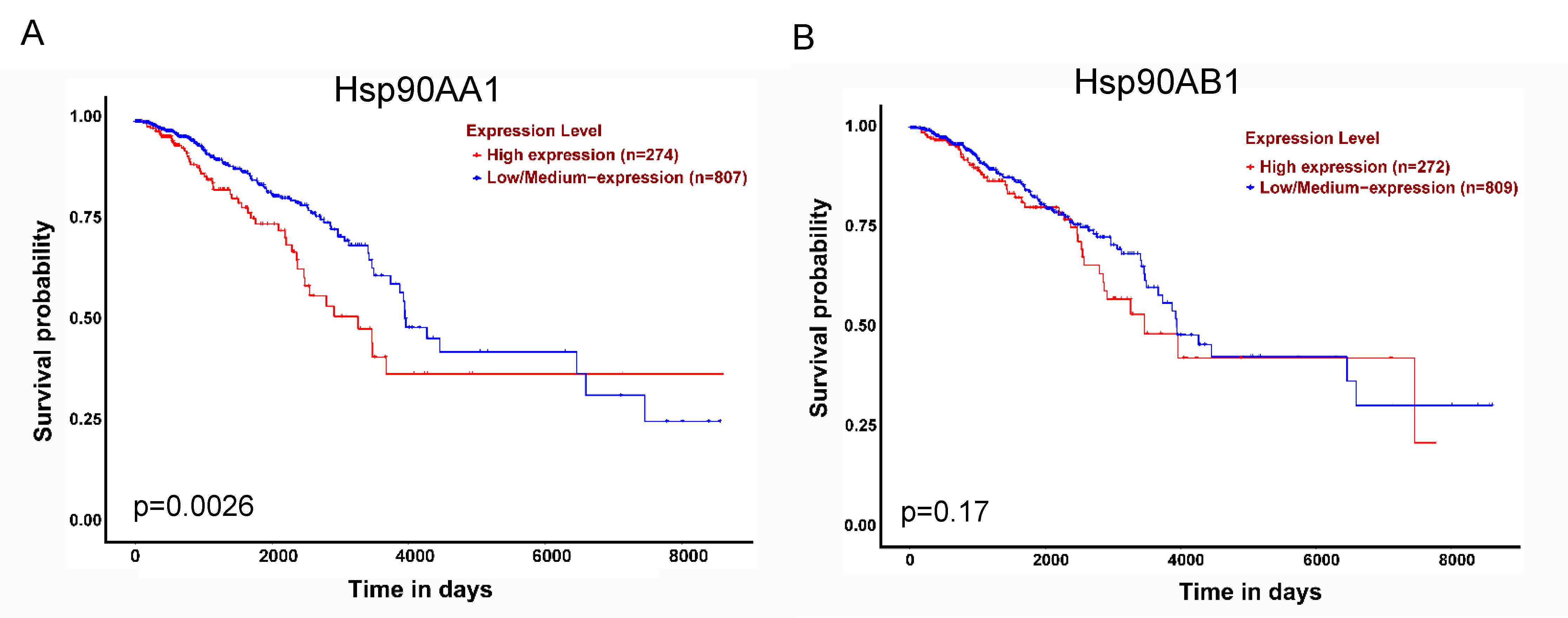

Supplement: Figure S1 — Cumulative survival analysis of breast cancer patients with low or high Hsp90AA1 (A) or Hsp90AB1 (B) expression. [file Image_1.TIFF]
