# Supplementary material for: BJ-B11, an Hsp90 Inhibitor, Constrains the Proliferation and Invasion of Breast Cancer Cells
Source: Front Oncol. 2019 Dec 18;9:1447. doi: 10.3389/fonc.2019.01447 (PMC6930179; doi:10.3389/fonc.2019.01447)
Supplement: Table S1 — Sample information. [file Table_1.DOCX]

Table S1 Sample information

| **Patient**  **NO.** | **Age**  **(Years)** | **Lymphatic**  **metastasis** | **Histological**  **type** | **Histological grade** | **Tumor**  **Size (cm)** | **ER** | **PR** | **HER-2** | **Ultrasound**  **BIRADS** |
| --- | --- | --- | --- | --- | --- | --- | --- | --- | --- |
| 1 | 53 | Yes | Breast invasive ductal carcinoma | Ⅱ | 2*2*1 | - | - | 3+ | Ⅳc |
| 2 | 44 | Yes | Breast invasive carcinoma | Ⅲ | 2*2*1.8 | 90%+ | 90%+ | 1+ | Ⅳc |
| 3 | 42 | No | Breast invasive ductal carcinoma | Ⅲ | 2*2*1.5 | - | - | 1+ | Ⅳa |
| 4 | 35 | Yes | Breast invasive ductal carcinoma | Ⅲ | 2.5*1*1 | 30%+ | 30%+ | 3+ | Ⅳb |
| 5 | 45 | Yes | Breast invasive carcinoma | Ⅱ | 2*1.5*1 | 80%+ | 90%+ | 2+ | Ⅳb |
| 6 | 62 | No | Breast invasive ductal carcinoma | Ⅱ | 6*2*1.5 | 80%+ | 4% | 3+ | Ⅳc |
| 7 | 46 | Yes | Breast invasive ductal carcinoma | Ⅱ | 1.6*1.5*0.8 | 60%+ | 70%+ | 1+ | Ⅴ |
| 8 | 32 | No | Breast invasive ductal carcinoma | Ⅲ | 2.2*2*2 | 90%+ | 65%+ | 1+ | Ⅳc |
| 9 | 45 | No | Breast invasive ductal carcinoma | Ⅱ | 2.5*2*1 | 30%+ | 20%+ | 3+ | Ⅳc |
| 10 | 51 | Yes | Breast invasive ductal carcinoma | Ⅲ | 1.6*1.5*1 | 85%+ | 90%+ | 1+ | Ⅳc |
| 11 | 51 | No | Breast invasive carcinoma | Ⅲ | 1.2*0.4*0.2 | - | - | - | Ⅳc |
| 12 | 72 | Yes | Breast invasive carcinoma | Ⅰ | 2*1.5*1 | 90%+ | 90%+ | 2+ | Ⅴ |
| 13 | 41 | Yes | Breast invasive ductal carcinoma | Ⅱ | 4*2*1.5 | 90%+ | 95%+ | - | Ⅳc |
| 14 | 58 | Yes | Breast invasive ductal carcinoma | Ⅲ | 2.5*2*1.5 | - | - | 3+ | Ⅳc |
| 15 | 51 | Yes | Breast invasive carcinoma | Ⅲ | 2*1.5*1.5 | - | - | 3+ | Ⅳc |
| 16 | 36 | Yes | Breast invasive ductal carcinoma | Ⅲ | 8.5*3.5*3 | 90%+ | 40%+ | 2+ | Ⅳc |
| 17 | 53 | No | Breast invasive carcinoma | Ⅱ | 1.3*1*0.5 | 80%+ | 90%+ | 1+ | Ⅳb |
| 18 | 38 | Yes | Breast invasive carcinoma | Ⅲ | 6*3.2*2 | 80% | 20%+ | 0 | Ⅳc |
| 19 | 49 | Yes | Breast invasive carcinoma | Ⅲ | 1.5*0.6*0.4 | 10%+ | 90%+ | 2+ | Ⅳc |
