# Supplementary material for: BJ-B11, an Hsp90 Inhibitor, Constrains the Proliferation and Invasion of Breast Cancer Cells
Source: Front Oncol. 2019 Dec 18;9:1447. doi: 10.3389/fonc.2019.01447 (PMC6930179; doi:10.3389/fonc.2019.01447)
Supplement: Table S2 — Primers used in this study. [file Table_2.DOCX]

Table S2 Primers used in this study

| Gene |  | Primer Sequence |
| --- | --- | --- |
| Human GAPDH | F | CCATCAATGACCCCTTCATTG |
|  | R | GACGGTGCCATGGAATTT |
| Human Hsp90AA1 | F | GAGCAGTACGCTTGGGAGTC |
|  | R | GTTTGGTCTTCTTTCAGGTGTAGG |
| Human Hsp90AB1 | F | AGACCCACTCCAACCGCAT |
|  | R | TCTCATCAGGAACTGCAGCAT |
| Human E-cadherin | F | AGGAGGTCTTTAAGGGGTCTGTCAT |
|  | R | TGGCGGCATTGTAGGTGTTC |
| Human Vimentin | F | AGATGGCCCTTGACATTGAGAT |
|  | R | TGGGTATCAACCAGAGGGAGT |
| Human Occludin | F | GGACTCTACGTGGATCAGTATTTG |
|  | R | TCATACCTGTCCATCTTTCTTCG |
