# Supplementary material for: BJ-B11, an Hsp90 Inhibitor, Constrains the Proliferation and Invasion of Breast Cancer Cells
Source: Front Oncol. 2019 Dec 18;9:1447. doi: 10.3389/fonc.2019.01447 (PMC6930179; doi:10.3389/fonc.2019.01447)
Supplement: Table S3 — Hsp family members upregulated in breast cancer tissue. [file Table_3.DOCX]

| **Protein description** | **Cancer/Normal Ratio** | **Regulated Type** | **Cancer/Normal**  **P value** | **Gene name** | **MW [kDa]** |
| --- | --- | --- | --- | --- | --- |
| Heat shock protein HSP 90-alpha OS=Homo sapiens GN=HSP90AA1 | 2.11 | Up | 1.11022E-16 | HSP90AA1 | 84.659 |
| Heat shock protein 75 kDa, mitochondrial OS=Homo sapiens GN=TRAP1 | 2.016 | Up | 0.00092084 | TRAP1 | 80.109 |
| Stress-70 protein, mitochondrial OS=Homo sapiens GN=HSPA9 | 1.922 | Up | 4.39648E-14 | HSPA9 | 73.68 |
| Heat shock protein beta-1 OS=Homo sapiens GN=HSPB1 | 1.825 | Up | 9.897E-09 | HSPB1 | 22.782 |
| 10 kDa heat shock protein, mitochondrial OS=Homo sapiens GN=HSPE1 | 1.708 | Up | 1.7256E-11 | HSPE1 | 10.932 |
| Heat shock 70 kDa protein 4 OS=Homo sapiens GN=HSPA4 | 1.7 | Up | 1.2576E-06 | HSPA4 | 94.33 |
| Heat shock cognate 71 kDa protein OS=Homo sapiens GN=HSPA8 | 1.598 | Up | 1.71374E-12 | HSPA8 | 70.897 |
| Heat shock protein HSP 90-beta OS=Homo sapiens GN=HSP90AB1 | 1.588 | Up | 0.00132318 | HSP90AB1 | 83.263 |
| 78 kDa glucose-regulated protein OS=Homo sapiens GN=HSPA5 | 1.444 | Up | 1.2058E-07 | HSPA5 | 72.332 |
| 60 kDa heat shock protein, mitochondrial OS=Homo sapiens GN=HSPD1 | 1.403 | Up | 1.3728E-06 | HSPD1 | 61.054 |
| Heat shock 70 kDa protein 1B OS=Homo sapiens GN=HSPA1B | 1.35 | Up | 2.6938E-06 | HSPA1B | 70.051 |

Table S3 Upregulated Hsp family in breast cancer
