# Supplementary material for: BJ-B11, an Hsp90 Inhibitor, Constrains the Proliferation and Invasion of Breast Cancer Cells
Source: Front Oncol. 2019 Dec 18;9:1447. doi: 10.3389/fonc.2019.01447 (PMC6930179; doi:10.3389/fonc.2019.01447)
Supplement: Table S4 — Differential Hsp90 expression in healthy and breast cancer tissues. [file Table_4.DOCX]

Table S4. Differential expression of Hsp90 in normal and breast cancer tissues

|  | n | Hsp90 expression | | Chi-square  Value | p value |
| --- | --- | --- | --- | --- | --- |
|  |  | High(%) | Low(%) |  |  |
| Breast cancer | 75 | 41 | 34 | 6.070 | 0.014* |
| Normal | 75 | 26 | 49 |  |  |

* Statistically significant (p<0.05)
