# Supplementary material for: BJ-B11, an Hsp90 Inhibitor, Constrains the Proliferation and Invasion of Breast Cancer Cells
Source: Front Oncol. 2019 Dec 18;9:1447. doi: 10.3389/fonc.2019.01447 (PMC6930179; doi:10.3389/fonc.2019.01447)
Supplement: Table S5 — Correlation between Hsp90 expression and clinicopathological characteristics. [file Table_5.DOCX]

Table S5. Correlation between Hsp90 expression and clinicopathological characteristics

|  | variables | Hsp90 expression | | total | χ^2^ | p value |
| --- | --- | --- | --- | --- | --- | --- |
|  |  | low | high |  |  |  |
| Age (year) |  |  |  |  | 1.000 | 0.317 |
|  | ≤56 | 41 | 30 | 71 |  |  |
|  | ＞56 | 33 | 34 | 67 |  |  |
| Grade |  |  |  |  | 4.999 | 0.025* |
|  | II | 58 | 39 | 97 |  |  |
|  | III | 16 | 25 | 41 |  |  |
| T stage |  |  |  |  | 1.109 | 0.292 |
|  | T1 | 27 | 29 | 56 |  |  |
|  | T2/T3 | 47 | 35 | 82 |  |  |
| N stage |  |  |  |  | 0.002 | 0.960 |
|  | N0 | 39 | 34 | 73 |  |  |
|  | N1N2N3 | 35 | 30 | 65 |  |  |
| TNM stage |  |  |  |  | 0.058 | 0.809 |
|  | Ι/II | 50 | 42 | 92 |  |  |
|  | III/IV | 24 | 22 | 46 |  |  |
| Relapse |  |  |  |  | 2.190 | 0.139 |
|  | Yes | 16 | 21 | 37 |  |  |
|  | No | 58 | 43 | 101 |  |  |

* Statistically significant (p<0.05)
