# Supplementary material for: BJ-B11, an Hsp90 Inhibitor, Constrains the Proliferation and Invasion of Breast Cancer Cells
Source: Front Oncol. 2019 Dec 18;9:1447. doi: 10.3389/fonc.2019.01447 (PMC6930179; doi:10.3389/fonc.2019.01447)
Supplement: Table S6 — Univariate and multivariate analyses of factors correlated with the overall survival of breast cancer patients. [file Table_6.DOCX]

Table S6. Univariate and multivariate analyses of the factors correlated with overall survival of breast carcinoma patients

| variables | Univariate analysis | | |  | Multivariate analysis | | |  |
| --- | --- | --- | --- | --- | --- | --- | --- | --- |
|  | HR | 95% CI | p value |  | HR | 95% CI | p value |  |
| expression | 2.747 | 1.243-6.075 | 0.013* |  | 2.866 | 1.257-6.533 | 0.012* |  |
| Grade | 2.377 | 1.130-4.998 | 0.022* |  | 1.586 | 0.716-3.517 | 0.256 |  |
| Age | 1.056 | 1.022-1.091 | 0.001** |  | 1.069 | 1.029-1.110 | 0.001** |  |
| T stage | 1.331 | 0.641-2.763 | 0.443 |  |  |  |  |  |
| N stage | 1.910 | 1.337-2.730 | 0.000** |  | 2.002 | 0.874-4.585 | 0.101 |  |
| TNM stage | 2.656 | 1.467-4.808 | 0.001** |  | 0.581 | 0.162-2.077 | 0.403 |  |
| Relapse | 0.088 | 0.037-0.209 | 0.000** |  | 0.112 | 0.040-0.311 | 0.000** |  |

Statistically significant: *p<0.05; **p<0.01.
